# Epidemiological changes in anti-glomerular basement membrane disease in Madrid in the context of the COVID-19 pandemic

**DOI:** 10.3389/fneph.2025.1667652

**Published:** 2025-09-10

**Authors:** Lina León-Machado, Gonzalo Sierra-Torres, Amir Shabaka, Clara Cases-Corona, Cristina Vega, Begoña Rivas, Diana Ruiz Cabrera, Gema Fernandez-Juarez

**Affiliations:** ^1^ Nephrology Department Hospital La Paz, Fundación para la Investigación Biomédica (Idipaz), Madrid, Spain; ^2^ Internal Medicine Department, Hospital Universitario Fundación Alcorcón, Madrid, Spain; ^3^ Nephrology Department, Hospital Universitario Fundación Alcorcón, Madrid, Spain

**Keywords:** vasculitis, goodpasture, incidence, COVID-19, renal survival

## Abstract

**Introduction:**

Recent studies in Europe have reported a rising incidence in anti-glomerular basement membrane (anti-GBM) disease, potentially linked to demographic shifts or environmental factors. This study aimed to assess temporal trends in incidence, clinical presentation, and outcomes of anti-GBM disease in two urban areas of Madrid over the past two decades.

**Materials and methods:**

We conducted a retrospective observational study of patients diagnosed with anti-GBM disease between 2006 and 2022 at two urban areas covering 884,000 residents in Madrid. Inclusion required confirmed anti-GBM antibodies with clinical manifestations. Incidence was calculated per 1,000,000 person-years. Data were analyzed across six time periods and compared pre- and post-COVID-19 onset.

**Results:**

A total of 26 cases were identified (mean age 52 ± 26 years; 54% female). Incidence increased from 1.13 cases per million persons-year before 2020, to 4.53 cases per million persons-year after 2020 (p<0.001). No differences were observed in demographic data or environmental exposures over time. Post-COVID-19 cases had lower serum creatinine at presentation (5.09 ± 4 *vs*. 8.7 ± 3.9 mg/dL, p=0.037), more pulmonary involvement (83.3% *vs*. 35.7%, p=0.039), and better 1-year renal survival (50% *vs*. 14.3%, p=0.049). Overall patient survival did not differ between groups.

**Conclusions:**

Incidence of anti-GBM disease has increased in Madrid, particularly after the COVID-19 pandemic. Improved renal survival appears linked to earlier diagnosis and management, rather than changes in environmental exposure. These findings highlight the importance of heightened clinical awareness for early detection and treatment of this aggressive disease.

## 1 Introduction

Anti-glomerular basement membrane (anti-GBM) disease is a rare autoimmune disease characterized by the presence of autoantibodies against the non-collagenous domain of the alpha-3 chain of type IV collagen, localized in glomerular and alveolar basement membranes (1), that mainly manifests with rapidly progressive glomerulonephritis and/or alveolar hemorrhage (2). The prognosis of anti-GBM disease with the current standard of therapy remains underwhelming, with a poor renal survival and high mortality rate (3). An early diagnosis and treatment are crucial to improve response to therapy and long-term prognosis (4).

Epidemiological changes in the incidence of anti-GBM disease have recently been identified in different European regions (5, 6), reporting an increased incidence in population-based studies in Ireland and Denmark, possibly due to demographic changes and exposure to environmental triggers (7, 8). Moreover, higher testing frequency and improved accessibility of diagnostic tests may have also contributed to this increase in disease incidence.

The aim of our study was to evaluate the changes in incidence of anti-GBM disease in the past two decades in two different urban areas in Madrid (North and southwest), to identify possible environmental triggers, and analyze temporal and within-area differences in presentation and outcome of the disease.

## 2 Materials and methods

We performed a retrospective observational study using data from the hospital coding databases and laboratory databases of two hospitals in two different urban areas in Madrid (North and Southwest Madrid) covering a population of 884,000 residents, between 2006 and 2022. This study was conducted in accordance with the Declaration of Helsinki. The study was approved by the local ethics committee (HULP code: PI-6197).

We collected data from patients diagnosed with anti-GBM disease with confirmed positive anti-GBM antibodies and renal and/or pulmonary manifestations. (**Supplementary Figure S1**). Anti-GBM antibodies were measured using standardized ELISA-based methods, and this methodology remained consistent across both centers and throughout the study period. The diagnostic threshold was set at ≥10 U/mL, in accordance with the manufacturer’s recommendations and prior studies. We excluded patients with antibody titers below 10 U/mL, as well as those with titers above this threshold but lacking clinical manifestations suggestive of anti-GBM disease (i.e., no renal involvement or pulmonary hemorrhage). Anti-GBM and ANCA antibodies were always tested concurrently in all patients with suspected rapidly progressive glomerulonephritis or pulmonary-renal syndrome.

For the temporal analysis of incidence, we divided the study period into two main intervals: pre-COVID-19 (January 2006 to February 2020) and post-COVID-19 (March 2020 to December 2022), aligning with the onset of the COVID-19 pandemic in Madrid.

Additionally, to explore more detailed temporal trends, we performed a secondary analysis dividing the entire study period into six intervals of three years each (2006–2008, 2009–2011, 2012–2014, 2015–2017, 2018–2020, and 2021–2022). This allowed a more granular assessment of incidence fluctuations over time while minimizing the potential exaggeration of random variations due to small case numbers.

Demographic and clinical data at presentation, including previous exposures to environmental factors (smoking, hydrocarbon exposure, infections, vaccinations), treatments received, and patient outcomes were systematically collected and analyzed.

Continuous variables are expressed as mean ± standard deviation or median (interquartile range), depending on their distribution. Categorical variables are presented as frequencies and percentages. The incidence rate of anti-GBM disease was calculated as the number of new cases per 1,000,000 person-years at risk. Corresponding 95% confidence intervals were estimated assuming a Poisson distribution. Comparisons between groups were carried out using the Chi-square test for categorical variables, Student’s *t*-test for normally distributed continuous variables, and the Mann–Whitney *U* test for non-parametric continuous variables. The date of diagnosis was defined as the date of the first positive anti-GBM antibody test. All included cases were incident cases. We ensured that survivors were only counted once and not re-included in subsequent denominators of the background population in following years, thereby preserving the integrity of incidence rate estimations. Incidence rates were calculated as the number of new cases per 1,000,000 person-years, using the actual population size for each year to adjust for any demographic changes over the study period.

An associated “previous infection” was defined as any infection occurring within 8 weeks prior to diagnosis of anti-GBM disease. Renal survival was defined as the time from diagnosis to the initiation of renal replacement therapy (dialysis or kidney transplantation). Patient survival was defined as the time from diagnosis to death from any cause. Patients were followed from the date of anti-GBM diagnosis to the occurrence of the relevant event (renal failure or death) or until the end of follow-up. Survival outcomes at 1 year were analyzed using binary logistic regression. All statistical analyses were performed using SPSS version 25.0 (IBM Corp., Armonk, NY, USA). A p-value < 0.05 was considered statistically significant.

## 3 Results

During the study period, 26 cases of anti-GBM disease were identified (incidence rate 2.94 per 1,000,000 persons per year); with a mean age of 52 ± 26 years at presentation, 27% of patients were double-seropositive for ANCA and anti-GBM, 42% presented only renal involvement, 4% with only pulmonary involvement, and 54% with both renal and pulmonary involvement. The clinical and histological characteristics and outcomes of each patient are detailed in **Supplementary Table S1**.

At presentation, median estimated glomerular filtration rate was 6.4 ml/min per 1.73 m^2^; median urine protein/creatinine ratio was 1.7 gr/gr, 19% of patients required mechanical ventilation and 65% required renal replacement therapy. After induction treatment, 13 patients (50%) remained on maintenance dialysis. After a median follow-up of 33 months, 46% of the patients died (**Table 1**). Renal and patient survival after 1 year was 30% and 73% respectively.

**Table 1:** Clinical characteristics and outcomes in anti-GBM cases presenting before and after the COVID-19 pandemic.

Variable	Total Cohort (n=26)	Before COVID-19 (n=14)	After COVID-19 (n=12)
Age at diagnosis, years^*^	52 ± 26	50.7 ± 30	57.9 ± 20.5
*Female sex, n (%)*	14 (53.8)	8 (57.1)	6 (50)
*Caucasian, n (%)*	25 (96.2)	13 (92.9)	12 (100)
*HTA, n (%)*	11 (42.3)	8 (57.1)	3 (25)
*Diabetes Mellitus, n (%)*	7 (26.9)	5 (35.7)	2 (16.7)
*Smoking, n (%)*	9 (34.6)	4 (28.6)	5 (41.7)
*Previous infection, n (%)*	3 (11.5)	2 (14.3)	1 (8.3)
*History of exposure to toxins, n (%):*	3 (11.5)	1 (7.1)	2 (16.7)
*Time from onset of initial symptoms to diagnosis of anti-GBM, days*	10 (6-24)	11 (7-22)	8 (6-46)
*Organ involvement, n (%)* *Kidney only* *Pulmonary only* *Kidney and pulmonary*	11 (42.3) 1 (3.8) 14 (53.8)	9 (64.3) 0 (0) 5 (35.7)	2 (16.7) 1 (8.3) 9 (75)
*AntiGBM antibody titer, UI/ml^#^*	116 [21.5-561]	139.5 [37.8–469]	65 [19.3–762.8]
*Double positive (ANCA+anti-GBM), n (%)* *ANCA subtype (MPO/PR3)*	7 (26.9) 1/6	5 (35.7) 0/5	2 (16.7) 1/1
Baseline Creatinine, mg/dl^*^	7 ± 4,4	8.7 ± 3.9	5.1 ± 4
*eGFR, ml/min/1,73m^2^*	6,4 [2-142]	5 [3-27]	9 [4-43]
*uPCR, g/g^#^*	1,7 [0-4]	1.3 [0.2-2.7]	1 [0.5-3]
Crescents, n (%)^*^	60.9 ± 31.9	79.5 ± 19	36 ± 29
*IS treatment, n (%)* *GC +CYC* *GC+CYC+RTX* *GC*	23 (88.5) 19 (73.1) 3 (11.5) 1 (3.8)	13 (92.9) 10 (71.4) 2 (14.3) 1 (7.1)	10 (83.3) 9 (75) 1 (8.3) 0 (0)
*Plasmapheresis, n (%)*	18 (69.2)	8 (57.1)	10 (83.3)
*Mechanical ventilation, n (%)*	5 (19.2)	1 (7.7)	4 (33.3)
*Renal replacement therapy at presentation, n (%)*	17 (65.4)	11 (78.6)	6 (50)
*Follow-up, months^#^*	33 [9-60]	54 [7-153]	31 [13-35]
*Maintenance dialysis, n (%)*	12 (46.2)	10 (71.4)	3 (25)
*Death, n (%)*	13 (50)	8 (61.5)	4 (36.4)

^*^Values are expressed as mean ± standard deviation. ^#^Values are expressed as median [interquartile range]

**Figure 1:**
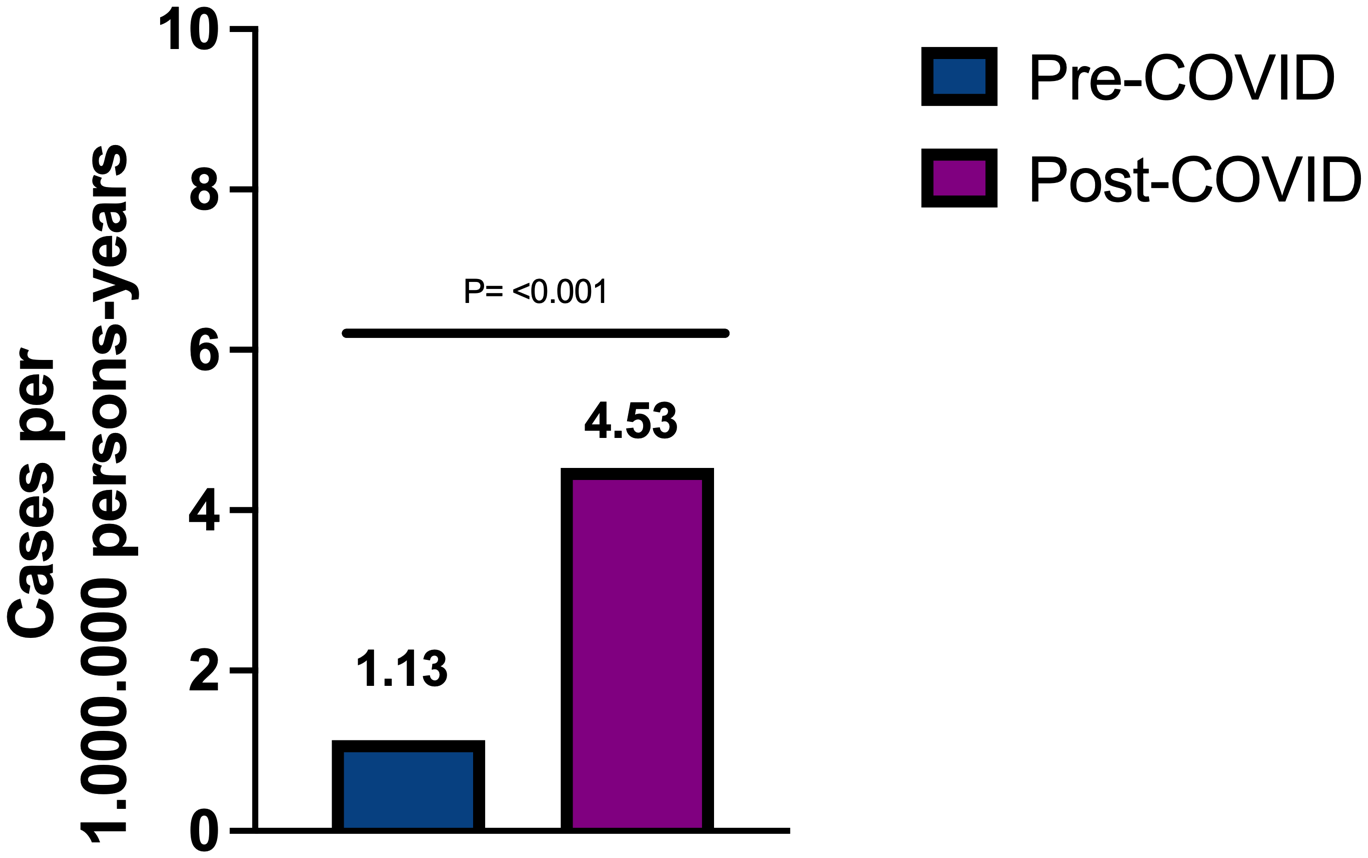
Incidence rate of anti-GBM disease in Madrid before and after COVID-19 pandemic.

We observed that the incidence rate significantly increased from 1.13 cases per 1,000,000 persons before March 2020 to 4.53 cases per 1,000,000 persons after the onset of the COVID-19 pandemic (incidence rate ratio= 4.0, CI 95% 1.85-8.65, p<0.001) (**Figure 1**). We observed an increase in the rate of anti-GBM antibody testing over time, particularly after 2017: from 183.7 tests per million person-years in 2014–2016, to 304.2 in 2017–2019, and 334.2 in 2020–2022. However, despite the increase in test frequency, the percentage of positive results rose significantly—from 0.68% before 2020 to 1.36% after 2020.

**Figure 2:**
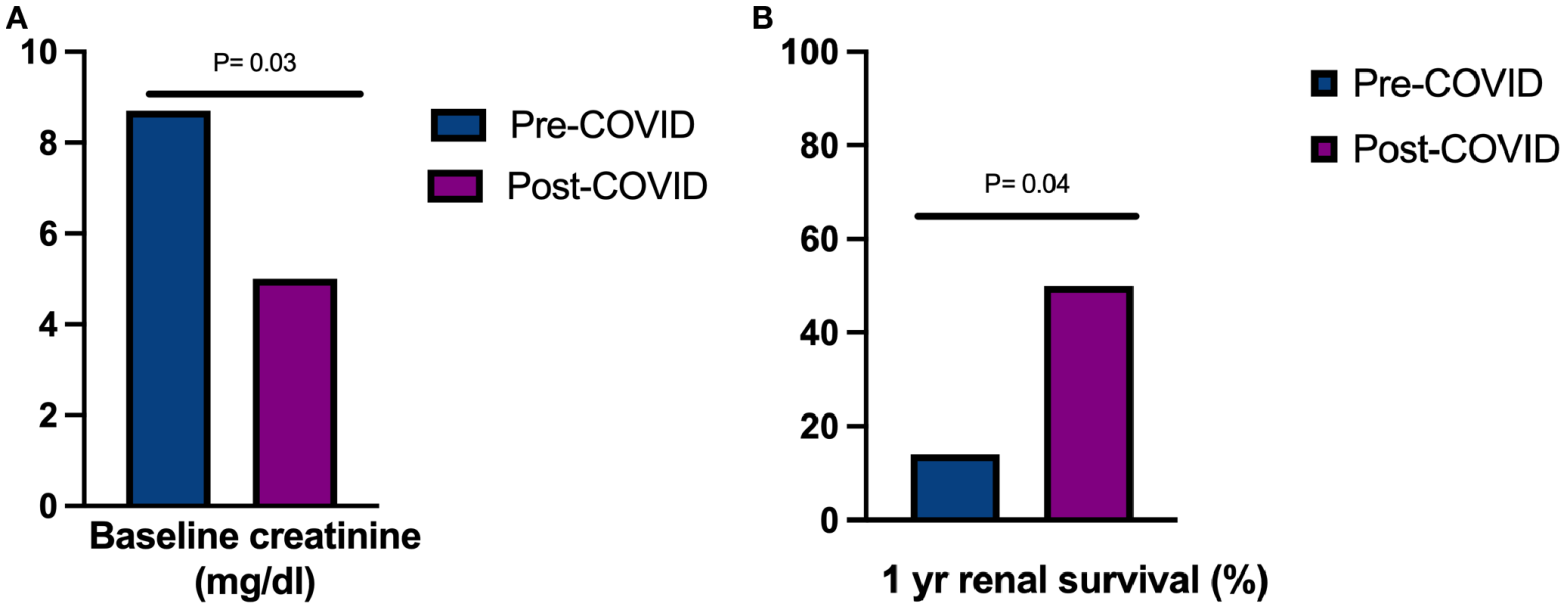
**(A)** Baseline serum creatinine at presentation in anti-GBM disease before and after COVID-19 pandemic, **(B)** Renal survival in anti-GBM disease in Madrid before and after the COVID-19 pandemic.

Regarding clinical presentation, serum creatinine at presentation was significantly lower in cases diagnosed after COVID-19 (5.09 ± 4 *vs*. 8.7 ± 3.9 mg/dl, p=0.037) (**Figure 2A**), but presented more frequently with pulmonary involvement (83.3% *vs*. 35.7%, p=0.039). There were no differences in proteinuria or hematuria. Fifteen out of the 26 patients (58%) underwent a kidney biopsy with sufficient material for diagnosis. Histologically, inflammatory findings were similar between cases diagnosed before and after the pandemic: all showed extracapillary proliferation and linear IgG deposits along the glomerular basement membrane, with no mesangial or endocapillary proliferation and no differences in direct immunofluorescence patterns. The only histological difference observed was in the percentage of glomeruli with cellular crescents, which was significantly higher in patients diagnosed before the pandemic (79.5 ± 19% *vs*. 36 ± 29%, p = 0.005), indicating more advanced disease at diagnosis. We noted that the 1-year renal survival rate increased from 14.3% in cases diagnosed before the onset of the COVID pandemic to 50% in cases diagnosed following COVID-19 (p=0.049) (**Figure 2B**). None of the cases were preceded by vaccination against SARS-CoV-2. Despite that, patients with anti-GBM following COVID-19 presented with more pulmonary involvement and more need of mechanical ventilation, survival analysis showed no differences in overall patient survival between both groups (log-rank χ^2^ = 0.09, p=0.759) (**Supplementary Figure S2**).

**Figure 3:**
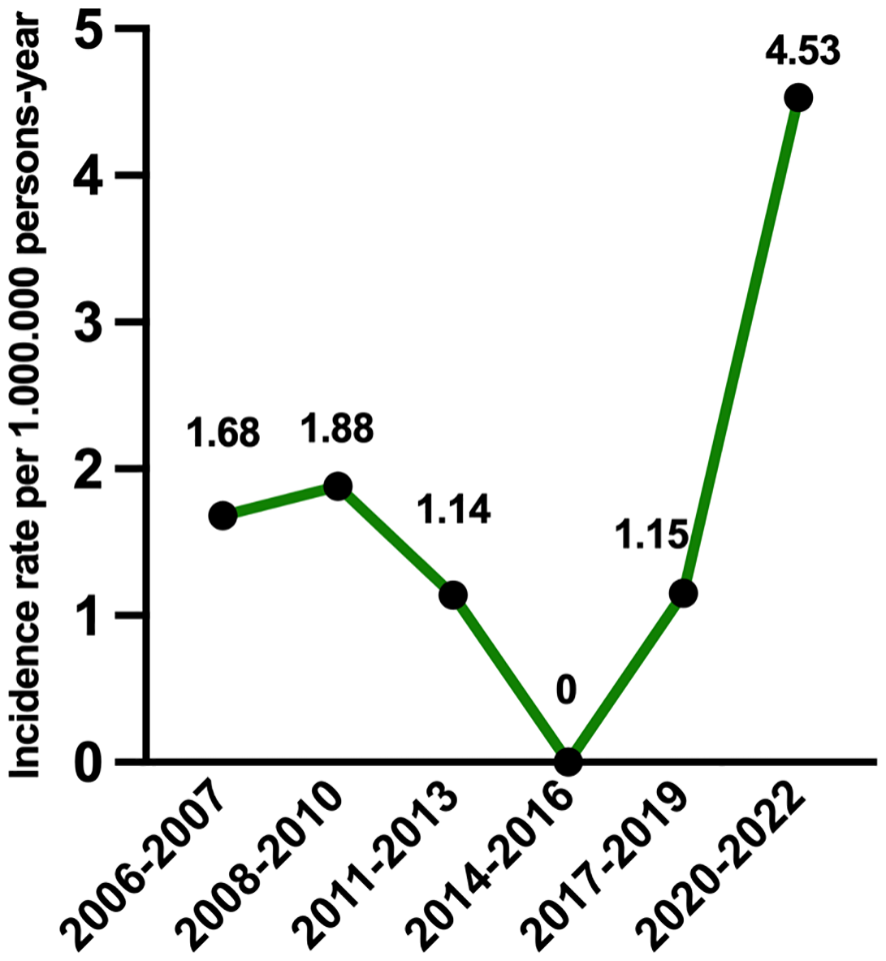
Incidence rate of anti-GBM disease in Madrid by time periods.

The incidence rate increased from 1.15 cases per 1,000,000 persons-year in 2017-2019, to 4.53 cases per 1,000,000 persons-year in 2020-2022 (**Figure 3**). We did not find differences within time periods in demographic data, previous exposure to environmental triggers, presence of double-seropositivity, or treatments received. However, we found that renal survival after 1 year improved from 0% in 2006–2007 to 25% in 2008-2010, 33% between 2011-2019, and to 66% in 2020-2022 (p=0.039). There were no differences in incidence, clinical presentation or outcomes between the different urban areas (**Supplementary Table S2**).

## 4 Discussion

This study explores the changes in incidence of anti-GBM disease in Madrid, observing an increasing incidence rate in the last two decades. The incidence rate increased particularly after March 2020, coinciding with the onset of the COVID pandemic.

The recent confirmation of spatial and temporal clustering of anti-GBM cases suggest that environmental factors may trigger the disease in susceptible persons (5). There has been an increased reporting of anti-GBM cases after COVID infection (7), similar to what had been previously described with an outbreak of Influenza A in Connecticut during the winter of 1971-1972 (8), which leads us to believe that COVID infection may play a role in the development of the disease. In our study we did not find differences in incidence within different urban areas in Madrid. However, this may be explained by the fact that COVID-19 incidence was similar in both areas. The increase in incidence cannot be explained by referral of patients from outside the catchment area, as all patients diagnosed with anti-GBM disease were residents within the study region.

Respiratory infections, including both Influenza and COVID-19, have been associated with the development of anti-GBM disease, likely through shared immunogenic mechanisms rather than viral family similarities. One proposed mechanism is molecular mimicry, in which microbial peptides resemble self-antigens and trigger cross-reactive immune responses. This has been demonstrated in murine models, where immunization with peptides mimicking an epitope of the α3 chain of type IV collagen led to the development of anti-GBM disease (9). Another plausible mechanism is direct tissue injury caused by viral infection, which may expose normally sequestered antigens such as the NC1 domain of the α3 chain of type IV collagen in the alveolar basement membrane, thus initiating or amplifying the autoimmune response (10, 11). Additionally, non-specific immune activation during acute viral infections can reduce the activation threshold of autoreactive lymphocytes, facilitating the breakdown of immune tolerance and triggering the production of anti-GBM antibodies (10, 11).

Among the 12 cases diagnosed after the onset of the COVID-19 pandemic, only one (Patient 23) had a documented SARS-CoV-2 infection prior to the diagnosis of anti-GBM disease. This infection occurred 7 months before diagnosis, and no clear association could be established. The time between infection and onset of anti-GBM disease in previously reported cases was after a maximum of 8 weeks, therefore it is improbable that in this case the development of disease had an association with infection. No cases of anti-GBM disease were diagnosed during the course of active COVID-19 infection. Despite the lack of direct evidence of infection in our series, we believe the observed increase in incidence following the onset of the pandemic may still reflect the influence of environmental or immunological factors during that period. Similar findings have been reported in other regions: for instance, studies from the United Kingdom (7) and India (12) also noted a rise in anti-GBM cases during the pandemic period, suggesting a potential shared environmental trigger. We have added contextual evidence from the literature to support this hypothesis. For example, Canney et al. (5) described clusters of anti-GBM disease associated with influenza A in genetically susceptible individuals (notably HLA-DRB1*1501 positive). Likewise, Prema et al. (12) reported a 68% increase in anti-GBM diagnoses during the COVID-19 pandemic, with serologic evidence of recent SARS-CoV-2 infection in several patients. A recurrence following COVID-19 infection has also been described (13).

Although some case reports suggest a temporal relationship between COVID-19 vaccination and anti-GBM disease (14–16), we found only one such case in our cohort, occurring seven weeks after the second vaccine dose. Therefore, vaccination does not appear to explain the overall increase in incidence in our study population. Patient 3 developed anti-GBM disease after receiving COVID-19 vaccination (2 months after the first dose and 1 month after the booster dose), presenting with pulmonary hemorrhage and severe AKI requiring RRT at onset. However, this patient had experienced non-specific symptoms (malaise, low-grade fever, myalgia) beginning approximately two weeks before vaccination. We cannot exclude the possibility of an undiagnosed SARS-CoV-2 infection prior to vaccination, or that the onset of anti-GBM disease occurred up to three months before its eventual diagnosis.

Demographic changes might also partly explain the observed differences in incidence rates. Patients diagnosed after March 2020 were slightly older (57 *vs*. 50 years) and included a higher proportion of females (57.1% *vs*. 50%), which may reflect underlying shifts in the background population. According to official census data, Madrid’s population has experienced significant aging over the study period: individuals under 40 years decreased by 21.3%, while those over 60 years increased by 36.2% between 2006 and 2022. In contrast, the sex distribution of the general population remained relatively stable (female sex: 51.6% in 2006 *vs*. 52.1% in 2022) (17). During the initial months following the pandemic declaration, mobility was significantly reduced due to lockdown measures and health restrictions, which likely limited patient referral patterns. Furthermore, all patients who tested positive for anti-GBM antibodies post-pandemic were residents of Madrid, minimizing the possibility that patients from neighboring regions artificially inflated our incidence estimates.

We observed an increase in the annual number of anti-GBM tests performed over time. Specifically, from 202.4 tests per million person-years before 2020 to 334.5 tests per million persons-year during 2020 and onward. Despite the increase in test frequency, the percentage of positive results rose significantly—from 0.68% before 2020 to 1.36% after 2020—suggesting that the rise in diagnosed cases is not solely attributable to increased testing, but likely reflects a real increase in incidence.

Our findings demonstrate a clear increase in the incidence of anti-GBM disease after the onset of the COVID-19 pandemic. While the rise in testing frequency and demographic shifts toward an older population may have influenced this trend, we cannot exclude a possible environmental influence related to the pandemic period, especially given the marked increase in the proportion of positive anti-GBM tests. Nevertheless, due to the observational nature of our study, no causal relationship can be established.

Patients diagnosed after March 2020 had better renal function at the time of presentation compared to those diagnosed before the onset of the COVID pandemic, which lead to better renal survival after 1 year of follow-up. This might be due to an increased awareness that leads to earlier serological testing in patients with rapidly progressive glomerulonephritis and in cases with alveolar hemorrhage, which translates into an earlier treatment. On the other hand, although cases described after COVID-19 presented with more pulmonary involvement and required more frequently mechanical ventilation, there were no differences in overall patient survival compared to cases described before the COVID pandemic. Recently, the development of imlifidase, an antibody-cleaving enzyme that degrades all IgG *in vivo* within few hours, has been held as a promising new therapeutic strategy to degrade anti-GBM antibodies promptly and thus improve renal and patient outcomes (18).

There are several limitations in this study that should be acknowledged. First, due to its retrospective nature, we cannot fully exclude the possibility that some cases of anti-GBM disease may have been preceded by infections or vaccinations that were not documented in the electronic health records. Furthermore, a direct association between confirmed COVID-19 infection and the development of anti-GBM disease cannot be established in our cohort, as not all patients had recorded SARS-CoV-2 PCR or serology results. While the observed increase in testing frequency and anti-GBM diagnoses coincided with the COVID-19 pandemic, these findings are observational in nature and do not establish causality. In our cohort, only one patient had a confirmed prior COVID-19 infection. Therefore, any association with the pandemic should be interpreted with caution. Additionally, we did not evaluate renal or patient survival beyond the first year after diagnosis. This decision was based on the limited number of patients remaining at risk beyond one year, as most reached the outcome of interest (either end-stage kidney disease or death) within the first 12 months. As a result, survival estimates beyond this point would lack statistical power and could be misleading.

In conclusion, the last few years have witnessed an increase in the incidence rate of anti-GBM disease in our population, particularly after the COVID-19 pandemic, but with improved renal survival. We did not find any changes in the rate of exposure to known environmental triggers within time periods. These findings suggest that the rising incidence may be attributable to an increased awareness and diagnosis of anti-GBM disease, which leads to a better renal survival.

## Data Availability

The raw data supporting the conclusions of this article will be made available by the authors, without undue reservation.

## Supplementary material

Supplementary Figure 1 Flowchart of patient selection

Supplementary Figure 2 Survival of patients with anti-GBM disease before and after COVID-19 pandemic

Supplementary Table 1 Summary of demographic, clinical, histological characteristics and outcomes of the cohort

Supplementary Table 2 Baseline clinical characteristics of patients by urban area in Madrid

## References

[1] Pusey CD. Anti-glomerular basement membrane disease. Kidney Int. (2003) 64:1535–50. doi: 10.1046/j.1523-1755.2003.00241.x, PMID: 12969182

[2] Jennette JC Falk RJ Bacon PA Basu N Cid MC Ferrario F. Revised international chapel hill consensus conference nomenclature of vasculitides. Arthritis Rheumatism. (2012) 65:1–11. doi: 10.1002/art.37715, PMID: 23045170

[3] Sánchez-Agesta M Rabasco C Soler MJ Shabaka A Canllavi E Fernández SJ. Anti-glomerular basement membrane glomerulonephritis: A study in real life. Front Med (Lausanne). (2022) 9:889185. doi: 10.3389/fmed.2022.889185, PMID: 35865174 PMC9295717

[4] McAdoo SP Pusey CD. Anti-glomerular basement membrane disease. CJASN. (2017) 12:1162–72. doi: 10.2215/CJN.01380217, PMID: 28515156 PMC5498345

[5] Canney M O’Hara PV McEvoy CM Medani S Connaughton DM Abdalla AA. Spatial and temporal clustering of anti-glomerular basement membrane disease. Clin J Am Soc Nephrol. (2016) 11:1392–9. doi: 10.2215/CJN.13591215, PMID: 27401523 PMC4974897

[6] Nelveg-Kristensen KE Madsen B McClure M Bruun N Lyngsø C Dieperink H. Age- and time-dependent increases in incident anti-glomerular basement membrane disease: a nationwide cohort study. Clin Kidney J. (2024) 17:sfad261. doi: 10.1093/ckj/sfad261, PMID: 38186880 PMC10768786

[7] Prendecki M Clarke C Cairns T Cook T Roufosse C Thomas D. Anti-glomerular basement membrane disease during the COVID-19 pandemic. Kidney Int. (2020) 98:780–1. doi: 10.1016/j.kint.2020.06.009, PMID: 32599088 PMC7318989

[8] Perez GO Bjornsson S Ross AH Amato J Rothfield N. A mini-epidemic of goodpasture’s syndrome. . Nephron. (1974) 13:161–73. doi: 10.1159/000180389, PMID: 4604010

[9] Li J-N Jia X Wang Y Xie C Jiang T Cui Z. Plasma from patients with anti-glomerular basement membrane disease could recognize microbial peptides. PloS One. (2017) 12:e0174553. doi: 10.1371/journal.pone.0174553, PMID: 28410377 PMC5391914

[10] McAdoo SP Pusey CD. Clustering of anti-GBM disease: clues to an environmental trigger? Clin J Am Soc Nephrol. (2016) 11:1324–6. doi: 10.2215/CJN.05580516, PMID: 27401526 PMC4974878

[11] Reggiani F L’Imperio V Calatroni M Pagni F Sinico RA. Goodpasture syndrome and anti-glomerular basement membrane disease. Clin Exp Rheumatol. (2023) 41:964–74. doi: 10.55563/clinexprheumatol/tep3k5, PMID: 36995324

[12] Prema KSJ Kurien A. Incidence of anti-glomerular basement membrane disease during the COVID-19 pandemic. Clin Kidney J. (2022) 15:180–1. doi: 10.1093/ckj/sfab204, PMID: 35028133 PMC8574335

[13] Winkler A Zitt E Sprenger-Mähr H Soleiman A Cejna M Lhotta K. SARS-CoV-2 infection and recurrence of anti-glomerular basement disease: a case report. BMC Nephrol. (2021) 22:75. doi: 10.1186/s12882-021-02275-4, PMID: 33639869 PMC7914035

[14] Tan MSH Choo JCJ Tan PH Kwek JL Lim CC Mok IY. Anti-glomerular basement membrane glomerulonephritis following COVID-19 infection without clinically evident pneumonia. Int Urol Nephrol. (2023) 55:1885–7. doi: 10.1007/s11255-023-03490-8, PMID: 36739354 PMC9899105

[15] Sacker A Kung V Andeen N. Anti-GBM nephritis with mesangial IgA deposits after SARS-CoV-2 mRNA vaccination. Kidney Int. (2021) 100:471–2. doi: 10.1016/j.kint.2021.06.006, PMID: 34119511 PMC8191282

[16] Coorey CP Phua E Chou A Shen Y Mather A. Anti-GBM Disease after Oxford-AstraZeneca ChAdOx1 nCoV-19 Vaccination: A Report of Two Cases. Case Rep Nephrol Dial. (2022) 12:234–7. doi: 10.1159/000525737, PMID: 36465571 PMC9710461

[17] Available online at: https://www.ine.es/jaxiT3/Tabla.htm?t=2881&L=0 (Accessed July 29th, 2025).

[18] Uhlin F Szpirt W Kronbichler A Bruchfeld A Soveri I Rostaing L. Endopeptidase cleavage of anti-glomerular basement membrane antibodies *in vivo* in severe kidney disease: an open-label phase 2a study. JASN. (2022) 33:829–38. doi: 10.1681/ASN.2021111460, PMID: 35260419 PMC8970456

