# Supplementary figures and images for: Epidemiological changes in anti-glomerular basement membrane disease in Madrid in the context of the COVID-19 pandemic
Source: Front Nephrol. 2025 Sep 10;5:1667652. doi: 10.3389/fneph.2025.1667652 (PMC12457157; doi:10.3389/fneph.2025.1667652)

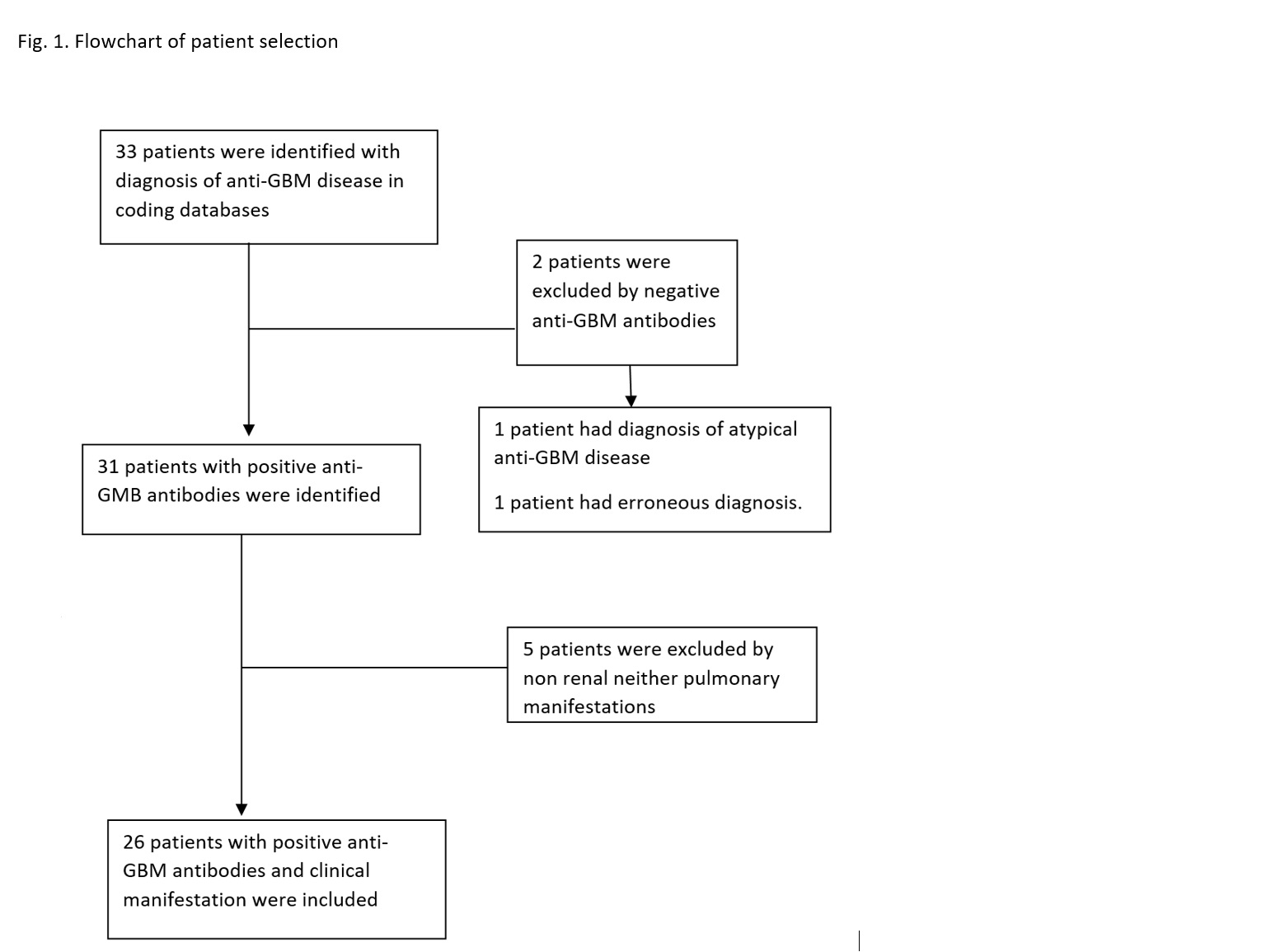

Supplement: Supplementary Figure 1 — Flowchart of patient selection [file Image1.jpeg]

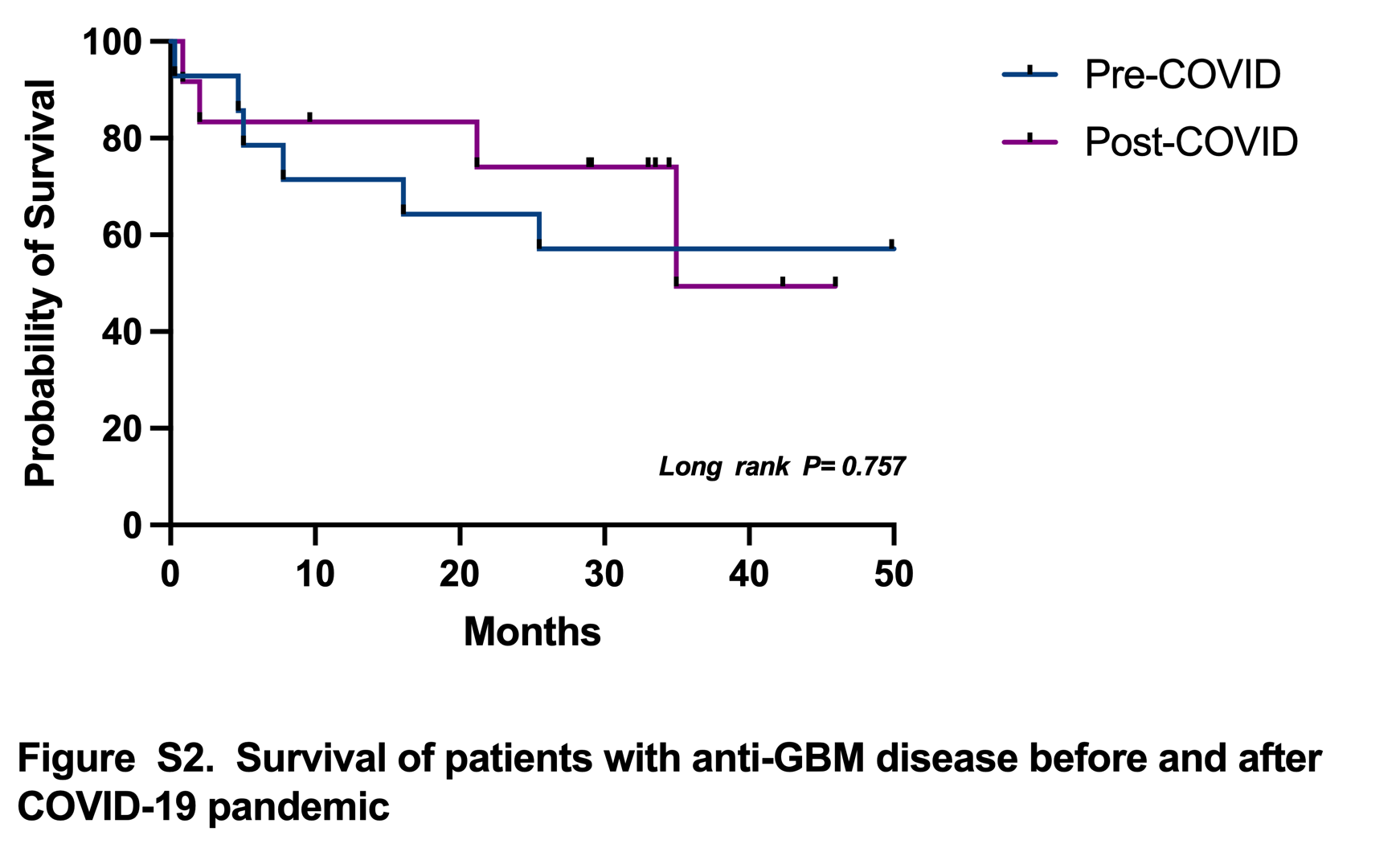

Supplement: Supplementary Figure 2 — Survival of patients with anti-GBM disease before and after COVID-19 pandemic [file Image2.tiff]
