# Supplementary material for: Epidemiological changes in anti-glomerular basement membrane disease in Madrid in the context of the COVID-19 pandemic
Source: Front Nephrol. 2025 Sep 10;5:1667652. doi: 10.3389/fneph.2025.1667652 (PMC12457157; doi:10.3389/fneph.2025.1667652)
Supplement: Supplementary Table 1 — Summary of demographic, clinical, histological characteristics and outcomes of the cohort [file Table1.docx]

**Table S1**. Summary of demographic, clinical, histological characteristics and outcomes of the cohort

| Patient | Area^+^ | Sex | Age | Year of diagnosis | Symptoms | Covid infection^*^ | Covid vaccine^*^ | Lung  Involvement | | Kidney involvement | Anti-GBM antibody titer  (u/ml) | ANCA overlap | | Kidney biopsy | | Treatment | Outcomes |
| --- | --- | --- | --- | --- | --- | --- | --- | --- | --- | --- | --- | --- | --- | --- | --- | --- | --- |
| 1 | North | F | 69 | 2018 | Low-grade fever, malaise | N/A | N/A | No | Cr: 17 mg/dl  eGFR 1.8 ml/min  microhematuria | | 204 | No | 65% Crescents, 3%  Glomerulosclerosis,  Linear GBM IgG | | | GC+CYC+PLEX | ESKD |
| 2 | North | F | 79 | 2020 | Low-grade fever, malaise | No | No | Yes confirmed in CT scan | Cr: 7.8 mg/dl  eGFR 4.4 ml/min  microhematuria | | >680 | No | 58% Crescents, No  Glomerulosclerosis,  Linear GBM IgG deposits | | | GC+CYC+RTX+ PLEX | ESKD |
| 3 | North | F | 73 | 2021 | Low-grade fever, malaise, dyspnea, hemoptysis | No | Yes | Yes confirmed in CT scan, required  MV | Cr: 7.6 mg/dl  eGFR 4.8 ml/min  microhematuria | | 790 | Yes  (MPO 92 UI/mL) | 9% Crescents, 78% Glomerulosclerosis, Linear GBM IgG deposits | | | GC+CYC+PLEX | ESKD |
| 4 | North | F | 38 | 2011 | Low-grade fever, malaise | N/A | N/A | No | Cr: 3.5 mg/dl  eGFR 15 ml/min  microhematuria | | 18 | Yes (MPO 30 UI/mL) | 50% Crescents, 22% Glomerulosclerosis,  Linear GBM IgG deposits | | | GC+CYC | Renal recovery |
| 5 | North | F | 84 | 2010 | Low-grade fever, malaise | N/A | N/A | Yes, confirmed in CT scan, oxygen supplementation needed | Cr: 12 mg/dl  eGFR 2.5 ml/min  Anuria | | 37 | No | 61% Crescents,  31% Glomerulosclerosis, Linear GBM IgG deposits | | | GC+CYC+PLEX | ESKD |
| 6 | North | M | 27 | 2006 | Low-grade fever, malaise, dyspnea | N/A | N/A | Yes confirmed in X-ray, oxygen supplementation needed | Cr: 12 mg/dl  eGFR 5 ml/min  microhematuria | | 1222 | No | 92% Crescents, 8% Glomerulosclerosis,  Linear GBM IgG deposits | | GC+CYC+ RTX+PLEX | | ESKD |
| 7 | North | F | 64 | 2020 | Hemoptysis, dyspnea | No | No | Yes confirmed in CT scan | Cr: 0.7 mg/dl  eGFR 91 ml/min  microhematuria | | 19 | No | No | | GC+CYC | | Recovery |
| 8 | North | F | 50 | 2021 | Malaise | No | No | No | Cr: 10 mg/dl  eGFR 4 ml/min  microhematuria | | 15 | No | 15% Crescents, No glomerulosclerosis  Linear GBM IgG deposits | | GC+CYC+ PLEX | | No |
| 9 | North | F | 75 | 2006 | Malaise | N/A | N/A | No | Cr: 9.4mg/dl  eGFR 3.6 ml/min  microhematuria | | 64 | Yes (MPO 76 UI/mL) | 30% Crescents, No glomerulosclerosis,  Linear GBM IgG deposits | | GC+CYC+ PLEX | | ESKD |
| 10 | North | M | 74 | 2020 | Dyspnea | No | No | Yes confirmed in CT scan | Cr: 0.8 mg/dl  eGFR 87 ml/min  microhematuria | | 17 | No | No | | GC+CYC | | Recovery |
| 11 | North | F | 15 | 2019 | Low-grade fever, malaise | N/A | N/A | No | Cr: 10 mg/dl  eGFR 4.8 ml/min  microhematuria | | 102 | Yes (MPO 86 UI/mL) | 85% Crescents, No glomerulosclerosis,  Linear GBM IgG deposits | | GC+CYC+ RTX+PLEX | | Renal recovery |
| 12 | North | M | 21 | 2021 | Dyspnea, hemoptysis | No | No | Yes confirmed in CT scan oxygen supplementation needed | Cr: 0.8 mg/dl  eGFR 140 ml/min  microhematuria | | 22 | No | No | | GC+CYC+ PLEX | | Recovery |
| 13 | North | M | 59 | 2022 | Dyspnea, malaise | No | No | No | Cr: 2 mg/dl  eGFR 35 ml/min  microhematuria | | 65 | No | 12% Crescents, 7% Glomeruloesclerosis,  Linear GBM IgG deposits | | GC+CYC+ PLEX | | Renal recovery |
| 14 | North | F | 84 | 2011 | Dyspnea, malaise | N/A | N/A | Yes confirmed in X-ray, oxygen supplementation needed | Cr: 5.6 mg/dl  eGFR 6.4 ml/min  microhematuria | | 38 | Yes (MPO 143UI/mL) | Insufficient material | | GC+CYC+ PLEX | | ESKD |
| 15 | North | M | 87 | 2008 | Low-grade fever, malaise, dyspnea | N/A | N/A | Yes confirmed in X-ray | Cr: 2 mg/dl  eGFR 29 ml/min  microhematuria | | 20 | No | No | | No Inmunosupressive treatment | | Recovery |
| 16 | North | F | 12 | 2008 | Low-grade fever, malaise, dyspnea | N/A | N/A | No | Cr: 6 mg/dl  eGFR 13 ml/min  microhematuria | | 288 | Yes (MPO 100 UI/mL) | 80% Crescents, No glomeruloesclerosis,  Linear GBM IgG deposits | | GC+CYC+ PLEX | | ESKD |
| 17 | North | M | 52 | 2021 | Low-grade fever, malaise, hemoptysis | No | No | Yes confirmed in CT scan oxygen supplementation needed | Cr: 6 mg/dl  eGFR 10 ml/min  microhematuria | | 10002 | Yes (PR3 61UI/mL) | 42% Crescents 42%, capsule rupture,  Linear GBM IgG deposits | | GC+CYC+ PLEX | | ESKD |
| 18 | SouthWest | F | 67 | 2007 | Low-grade fever, malaise | N/A | N/A | No | Cr: 11 mg/dl  eGFR 3 ml/min  microhematuria | | 547 | No | 100% Crescents, No glomerulosclerosis  Linear GBM IgG deposits | | GC+CYC+ PLEX | | ESKD |
| 19 | SouthWest | M | 61 | 2008 | malaise | N/A | N/A | No | Cr: 8 mg/dl  eGFR 6.4 ml/min  microhematuria | | 443 | No | No | | GC+CYC+ PLEX | | ESKD |
| 20 | SouthWest | M | 59 | 2009 | Low-grade fever, malaise, dyspnea | N/A | N/A | Yes | Cr: 8.7 mg/dl  eGFR 6 ml/min  microhematuria | | 149 | No | No | | No inmunosupressive treatment | | No |
| 21 | SouthWest | M | 55 | 2011 | Low-grade fever, malaise | N/A | N/A | No | Cr: 8.3 mg/dl  eGFR 6.6 ml/min  microhematuria | | 600 | No | 100% Crescents, No glomerulosclerosis,  Linear GBM IgG deposits | | GC+CYC+ PLEX | | ESKD |
| 22 | SouthWest | M | 76 | 2017 | Malaise | N/A | N/A | No | Cr: 6.7 mg/dl  eGFR 7.4 ml/min  microhematuria | | 130 | No | 60% Crescents, No glomerulosclerosis,  Linear GBM IgG deposits | | GC+CYC | | ESKD |
| 23 | SouthWest | M | 74 | 2021 | Malaise, dyspnea, hemoptysis | No | No | Yes,  Required MV | Cr: 1 mg/dl  eGFR 90 ml/min  no microhematuria | | 20 | No | No | | GC+CYC+ PLEX | | Death (respiratory failure) |
| 24 | SouthWest | M | 57 | 2021 | Malaise, dyspnea, hemoptysis | No | No | Yes, confirmed by CT scan and BAL  Required MV | Cr: 13 mg/dl  eGFR 3.8 ml/min microhematuria | | 307 | No | No | | GC+CYC+ PLEX | | ESKD, Death (respiratory failure) |
| 25 | SouthWest | F | 16 | 2021 | Malaise, dyspnea, hemoptysis | No | No | Yes, confirmed in CT scan and BAL | Cr: 0.7 mg/dl  eGFR 90 ml/min microhematuria | | 65 | No | No | | GC+CYC+ PLEX | | Recovery |
| 26 | SouthWest | F | 73 | 2022 | Dyspnea, hemoptysis | No | No | Yes  required MV | Cr: 10 mg/dl  eGFR 3.4 ml/min  Anuria | | 1000 | No | No | | GC+CYC+ PLEX | | ESKD, death |

Cr, Serum creatinine; CT, computed tomography; CYC, cyclophosphamide; eGFR, estimated glomerular filtration rate; ESKD, end stage kidney disease; F, female;

GBM, glomerular basement membrane; GC, glucocorticoids; M, male; MV, mechanical ventilation; PLEX, plasma exchange;

^+^ Area of Madrid

^*^COVID infection or COVID vaccine no more than 8 weeks prior to diagnosis of Anti-GBM disease
