# Supplementary material for: Epidemiological changes in anti-glomerular basement membrane disease in Madrid in the context of the COVID-19 pandemic
Source: Front Nephrol. 2025 Sep 10;5:1667652. doi: 10.3389/fneph.2025.1667652 (PMC12457157; doi:10.3389/fneph.2025.1667652)
Supplement: Supplementary Table 2 — Baseline clinical characteristics of patients by urban area in Madrid [file Table2.docx]

**Table S2**. Baseline clinical characteristics of patients by urban area in Madrid

| **Variable** | **North Madrid**  **N:17** | **Southwest Madrid**  **N:9** |
| --- | --- | --- |
| Age at diagnosis (yr) | 57±25 | 60±18 |
| Female sex, n (%) | 11 (65) | 3 (33) |
| Caucasian, n (%) | 16 (94) | 9 (100) |
| Hypertension, n (%) | 6 (35) | 5 (55) |
| Diabetes Mellitus, n (%) | 4 (23) | 3 (33) |
| Smoking, n (%) | 5 (29) | 4 (44) |
| Previous infection, n (%) | 2 (12) | 1 (11) |
| History of exposure to toxins, n (%): | 2 (12) | 1 (11) |
| duration from onset of initial symptoms to diagnosis of anti-GBM, days | 11 (5-30) | 8 (6-13) |
| Organ involvement, n (%)  Kidney  Pulmonary  Kidney and pulmonary | 7 (41)  0 (0)  10 (59) | 4 (44)  1 (11)  4 (44) |
| ANCA and Anti-GBM, n (%) | 7 (41)  1/6 | 0 |
| Baseline Creatinine, mg/dl | 7±5 | 8±4 |
| eGFR, ml/min/1,73m^2^ | 6 [4-32] | 6 [4-37] |
| uPCR, g/g | 1 [0,3-2] | 2 [0,7-4] |
| Crescents, n (%): | 52±29.9 | 93.3±11.5 |
| IS treatment, n (%)  GC +CYC  GC+CYC+RTX  GC | 15 (88)  11 (65)  3 (18)  1( 6) | 8 (89)  8 (89)  0(0)  0 (0) |
| Plasmapheresis, n (%) | 12 (70) | 6 (67) |
| Mechanical ventilation, n (%) | 2 (12) | 3 (33) |
| Renal replacement therapy at presentation, n (%) | 11 (65) | 6 (67) |
| Follow-up ,months | 35 [22-109] | 21[1,4-46] |
| Maintenance dialysis, n (%) | 9 (53) | 4 (44) |
| Death, n (%) | 5 (29) | 7 (78) |

Data are presented as n (%), mean ± standard deviation, or median (interquartile range).

Abbreviations: ANCA: anti-neutrophil cytoplasmic antibodies, Anti-GBM: anti-glomerular basement membrane antibodies, CF: cyclophosphamide, eGFR: estimated glomerular filtration rate, GC:glucocorticoids, IS: Immunosupressive, RTX: rituximab, uPCR: urine protein/creatinine ratio;
